# Reconstruction of extensive thoracolumbar defect with perforator-based double Keystone Island flap

**DOI:** 10.1093/jscr/rjad505

**Published:** 2023-09-08

**Authors:** Rhea M Mužar, Domagoj Eljuga, Josip Jaman, Zlatko Vlajčić, Rado Žic

**Affiliations:** Department of Plastic, Reconstructive and Aesthetic Surgery, University Hospital “Dubrava”, 10'000 Zagreb, Croatia; Department of Plastic, Reconstructive and Aesthetic Surgery, University Hospital “Dubrava”, 10'000 Zagreb, Croatia; Department of Plastic, Reconstructive and Aesthetic Surgery, University Hospital “Dubrava”, 10'000 Zagreb, Croatia; Department of Plastic, Reconstructive and Aesthetic Surgery, University Hospital “Dubrava”, 10'000 Zagreb, Croatia; Department of Plastic, Reconstructive and Aesthetic Surgery, University Hospital “Dubrava”, 10'000 Zagreb, Croatia

**Keywords:** dermatofibrosarcoma protuberans, reconstruction, Keystone Island flap, plastic surgery, oncology

## Abstract

Sarcomas represent 1% of malignancies in adult population; thereby dermatofibrosarcoma protuberans is found in 1%–2% of all cases. A surgical approach in oncologic treatment is the standard of care; therefore, important is an extensive resection to achieve clear margins and prevent recurrence. Herein we report a case of recurrent dermatofibrosarcoma protuberans in the thoracolumbar region. As the adequate resection was to cause a huge defect, we have made a reconstruction plan for coverage, using a perforator-based double Keystone Island flap (Type III). The method was chosen as a reliant solution with low complication rates and without need for complex flap designs thereby giving good functional and esthetic results. Main aim of the case report was to show that Keystone Island flaps present a simple and technically straightforward method with low complication rates and good results.

## Introduction

Dermatofibrosarcoma protuberans is a very rare and locally aggressive malignant cutaneous soft-tissue sarcoma with high probability of local recurrence [1]. Complete surgical resection with wide free margins is the only treatment that can minimize local recurrence rates [2]. Due to the extensive defects that are usually present after resection, primary closure is often not possible; therefore, different individual reconstructive options should be evaluated.

## Case report

**Figure 1:**
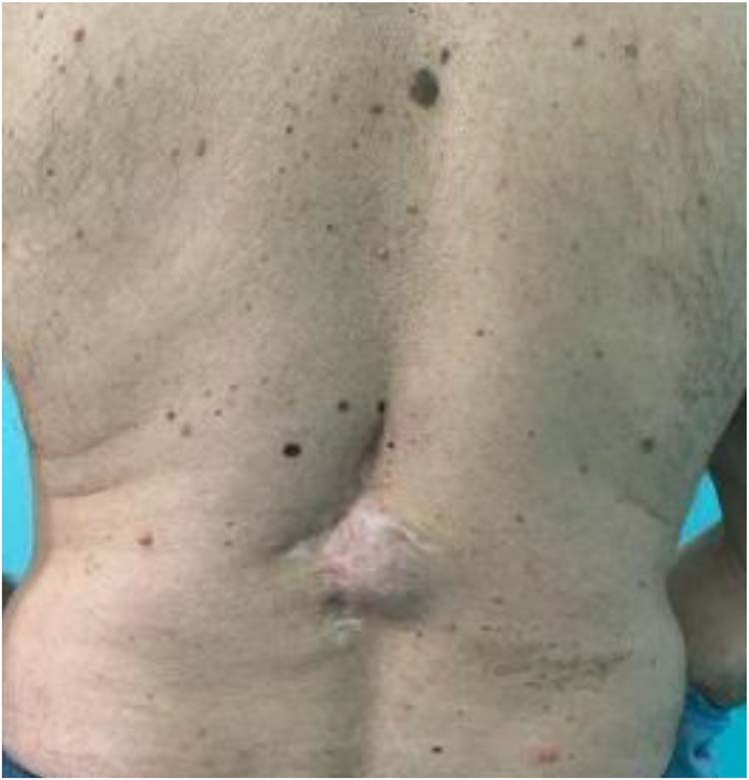
Preoperative local finding.

**Figure 2:**
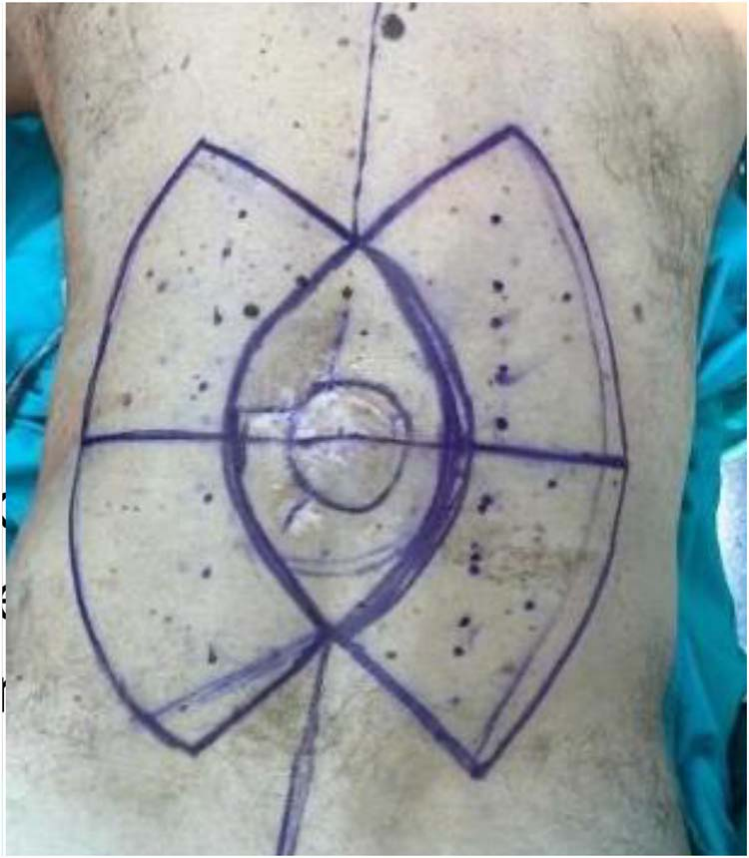
Preoperative markings.

**Figure 3:**
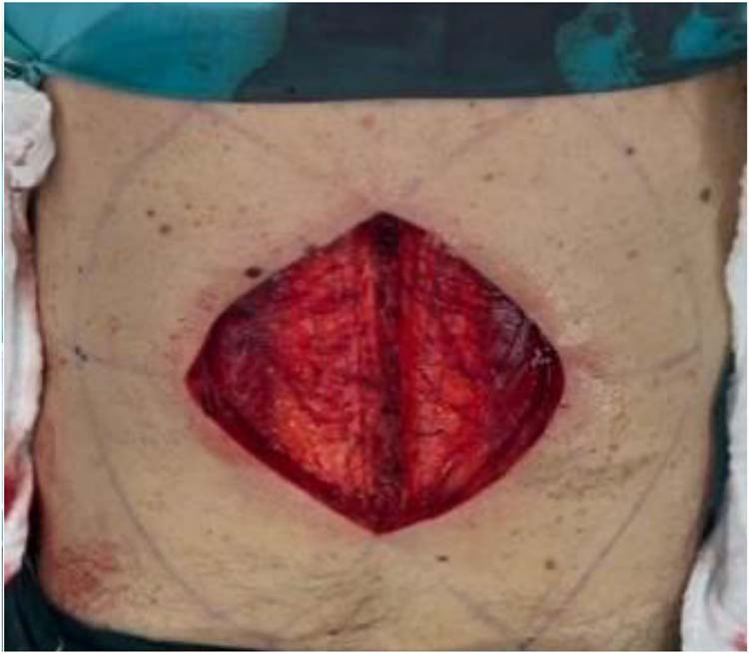
Intraoperative extent of the defect.

**Figure 4:**
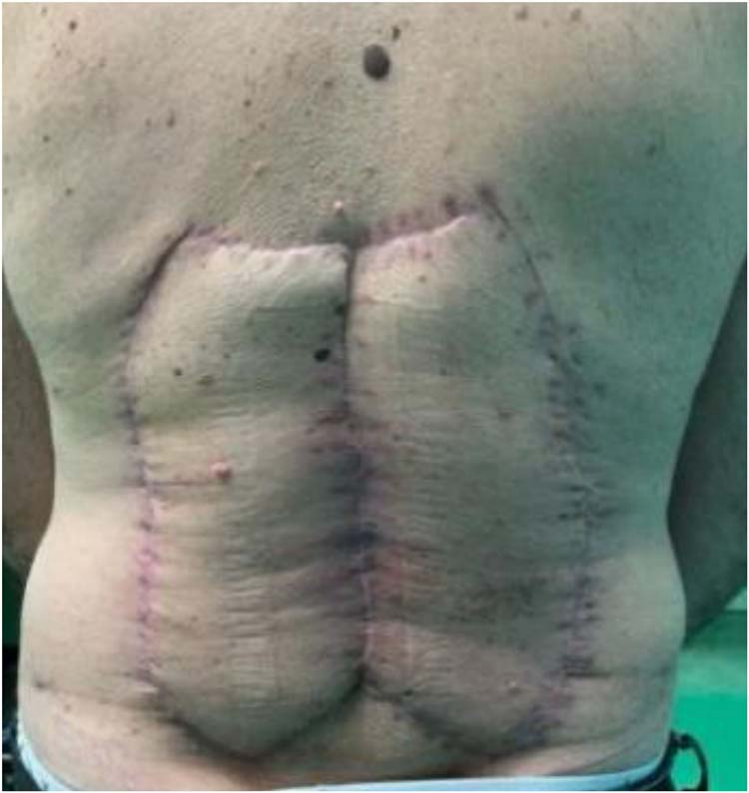
Local findings 1 month after surgery.

A 63-year-old male patient was admitted to the Department of Plastic and Reconstructive surgery in University Clinical Hospital Dubrava for surgical treatment of a recurrent soft tissue sarcoma in the thoracolumbar region. Two years prior to this referral, he had a biopsy at another County Hospital when a subcutaneous tumor was resected. Pathohistological evaluation showed it was dermatofibrosarcoma protuberans. After that, a wider excision was undertaken with narrow free margins and primary closure of the wound. In the follow-up period, 18 months after the procedure, during a clinical checkup, a suspect mass appeared in the scar where resection was performed (Fig. 1). Computed tomography scan and ultrasound showed a 3.6 × 2.6 cm heterogeneous mass with infiltration of the erector spinae muscle. A biopsy was performed and showed monomorphic mesenchymal tissue positive for CD34, corresponding to a recurrence of the primary tumor. Metastases were not detected. Looking at the clinical appearance of the patient as well as his overall good health, we planned a wide resection. We anticipated a large defect after resection in the thoracolumbar area, which was needed for clear resection margins; therefore, a reconstruction of soft tissue coverage was needed. We decided to use a double Keystone Island flap. The pattern was marked preoperatively in a prone position (Fig. 2). Skin incisions were performed based on the preoperative markings with respect to the lumbar perforators. The mass was broadly resected, with partial resection of the erector spinae muscle down to the spinous process of L1 (Fig. 3). The tumor was sent for pathohistological analysis. With blunt dissection, the strongest perforating vessel on each side was found, and fasciocutaneous flaps were elevated, preserving vascular integrity. As each of the flaps was only dependent on one perforator, we achieved maximal mobility. Surrounding tissue was advanced and connected in the vertebral midline; thereby, the defect was closed with minimal tension. Postoperatively, the patient presented well at the ward and was discharged in good health with irritation-free wounds. Two weeks after surgery, the flaps were vital without signs of seroma or infection. After 1 month, the suturing material was completely removed, showing full acceptance of the flaps with well-healed wounds (Fig. 4).

Pathohistological analysis showed complete resection of the tumor mass with free margins. Further oncological care for the patient was induced. Additionally, the patient will be checked up every 6 months in the postoperative period.

## Discussion

The Keystone Island flap was first described in 2003 [3]. It is a perforator-based island flap that is applicable on all body parts and used as a reliant technique of soft tissue defect closure, avoiding more complex flap designs [4, 5]. It shows better results and lower complication rates than skin grafts [6, 7]. As there are four types (Behan’s Classification) of flap design, there are many possibilities for reconstruction [3, 5]. The flap is designed with a 1:1 flap-to-defect ratio and is based on a perforator vessel either raised from musculocutaneous or fasciocutaneous origin. Orientation should be made on the axis of the corresponding perforator and his vascular territory. Advancement is made in a V-Y manner. Increased mobility with extensive undermining away from the vascular supply can be achieved. Complications such as partial or total flap loss are rare, measuring between 5% and 10% [8]. Morbidity is reduced as the design of the flap allows minimal tension at closing, vascular supply is provided, especially in the intercostal, lumbar, and gluteal regions dominant vessels are well-known, thereby perfusion is secured. Additionally, esthetic results are good as the flap has no extensive tissue bulk [3, 7]. We showed that a structured surgical treatment plan is crucial for meeting oncological requirements as well as functional and esthetic aspects in extensive soft tissue resections. Important factors thereby are considering the size of the defect, the definition of a donor site with adequate vascular status, the overall health of the patient, and choosing the right technique.

We show, with respect to our case, that the reconstruction of wide soft tissue defects using a perforator-based Keystone Island flap is a simple and technically straightforward method with low complication rates. For the right indication, it should be more often considered as an option for treatment by plastic surgeons practicing oncological surgery.

## Data Availability

The authors confirm that the data supporting the findings of this study are available within the article and its supplementary materials.

## References

[1] Llombart B, Serra C, Requena C. et al. Guidelines for diagnosis and treatment of cutaneous sarcomas: dermatofibrosarcoma protuberans. Actas Dermosifiliogr 2018;109:868–77. 30539729 10.1016/j.ad.2018.05.006

[2] Demetri GD, Baker LH, Beech D. et al. Soft tissue sarcoma clinical practice guidelines in oncology. J Natl Compr Canc Netw 2005;3:158–94. 19817028 PMC5788173

[3] Behan F, Sizeland A, Porcedu S. et al. Keystone Island flap: an alternative reconstructive option to free flaps in irradiated tissue. ANZ J Surg 2006;76:407–13. 16768705 10.1111/j.1445-2197.2006.03708.x

[4] Hessam S, Sand M, Bechara FG. The keystone flap: expanding the dermatologic surgeon's armamentarium. J Dtsch Dermatol Ges 2015;13:70–2. 25640508 10.1111/ddg.12542

[5] Gómez OJ, Barón OI, Peñarredonda ML. Keystone flap: overcoming paradigms. Plast Reconstr Surg Glob Open 2019;7:e2126. 10.1097/GOX.0000000000002126. 31044108 PMC6467614

[6] Le Guern A, Wiart T, Modiano P, Lebas D. The keystone flap and its simplified version for malignant skin tumor defects of the lower limbs: a review of 25 cases. Ann Dermatol Venereol 2021;148:241–5. 34756482 10.1016/j.annder.2021.04.006

[7] Behan FC . The keystone design perforator island flap in reconstructive surgery. ANZ J Surg 2003;73:112–20. 12608972 10.1046/j.1445-2197.2003.02638.x

[8] Formentin C, de Andrade EJ, Matias LG. et al. Using the keystone design perforator island flap in large myelomeningocele closure. Neurosurg Focus 2019;47:E19. 10.3171/2019.7.FOCUS19383 31574473

